# Nationwide Trends in Arthroscopic Knee Surgery and ACL Reconstruction in Romania, 2017–2023: Insights from a Seven-Year Health System Analysis

**DOI:** 10.3390/life15111734

**Published:** 2025-11-12

**Authors:** Gloria Alexandra Tolan, Cris Virgiliu Precup, Bogdan Uivaraseanu, Delia Mirela Tit, Gabriela S. Bungau, Andrei-Flavius Radu, Cristian George Furau

**Affiliations:** 1 Multidisciplinary Doctoral School, “Vasile Goldis” Western University of Arad, 310414 Arad, Romania; gloria.tolan@yahoo.de; 2 Department of Life Sciences, “Vasile Goldis” Western University of Arad, 310414 Arad, Romania; precupcris@yahoo.com; 3 Department of Surgical Disciplines, Faculty of Medicine and Pharmacy, University of Oradea, 410073 Oradea, Romania; 4 Doctoral School of Biological and Biomedical Sciences, University of Oradea, 410087 Oradea, Romania; gbungau@uoradea.ro; 5 Department of Pharmacy, Faculty of Medicine and Pharmacy, University of Oradea, 410028 Oradea, Romania; 6 Department of Psycho-Neurosciences and Recovery, Faculty of Medicine and Pharmacy, University of Oradea, 410073 Oradea, Romania; andreiflavius.radu@uoradea.ro; 7 Department of Pathophysiology, Faculty of Medicine, “Vasile Goldis” Western University of Arad, 310414 Arad, Romania; furau.cristian@uvvg.ro

**Keywords:** arthroscopy, knee, ACL reconstruction, meniscectomy, epidemiology, health system, regional disparities, Romania

## Abstract

Arthroscopic knee surgery represents a cornerstone of modern orthopedic practice, yet nationwide data from Eastern Europe remain scarce. This study provides the first comprehensive assessment of arthroscopic knee procedures in Romania over a seven-year period (2017–2023), focusing on anterior cruciate ligament (ACL) reconstruction trends and related interventions. Using national hospital discharge data, all arthroscopic knee procedures were identified and analyzed by year, sex, age group, region, and hospital type. A total of 76,804 procedures were recorded, including 26,888 reconstructions (O15301/O15303) and 29,979 meniscectomies (O13404). ACL reconstructions increased from 1560 cases in 2017 (7.9/100,000 inhabitants) to 1865 in 2023 (9.8/100 k), with a marked decline in 2020 (5.3/100 k) due to the COVID-19 pandemic and full recovery thereafter. Men predominated in ACL reconstructions (74%; 8226 males vs. 2854 females), whereas meniscectomy peaked in middle-aged adults (50–54 years: 48.7/100 k). Surgical activity was highly centralized, with five counties performing over two-thirds of all ACL reconstructions. Approximately 89% of procedures were conducted in public hospitals. These findings reveal substantial progress but also persistent regional and demographic inequities. Strengthening access, standardizing indications for degenerative meniscal surgery, and establishing a national ACL registry could support equitable, evidence-based advancement of arthroscopic care in Romania.

## 1. Introduction

Anterior cruciate ligament (ACL) injuries are among the most common knee injuries in young and physically active individuals. These lesions are frequently associated with meniscal and cartilage damage [1,2]. Delaying surgical reconstruction significantly increases the risk of additional intra-articular damage [3]. In acute ACL injuries, severe cartilage damage has been observed in 16–46% of patients, with partial meniscectomy strongly correlated with an increased risk of subsequent chondral deterioration compared to intact or repaired menisci [4].

Surgical reconstruction, typically performed arthroscopically, remains the standard of care for restoring anterior–posterior and rotational stability of the knee in physically active patients [5]. While ACL autograft reconstruction using hamstring or patellar tendon grafts has long been the gold standard, there is a growing interest in alternative grafts, such as the quadriceps tendon, which has recently regained popularity [6]. Moreover, primary arthroscopic ACL repair has shown promising short- to mid-term outcomes, with comparable failure rates, complication rates, and patient satisfaction to those of traditional autograft reconstruction [7].

Thanks to advances in arthroscopic surgery, minimally invasive approaches now allow surgeons to address not only ACL reconstruction but also associated injuries such as meniscal tears, cartilage lesions, and synovial damage. Arthroscopic meniscectomy (partial or total resection of a torn meniscus), meniscal repair, synovectomy, and ligament reconstructions (for ACL, posterior cruciate ligament (PCL), and other structures) are now widely practiced worldwide [8,9].

Over the past decades, many countries have reported a steady rise in the incidence of ACL reconstructions. In South Korea, population-level data from 2011 to 2018 show a significant rise in surgeries among adolescents aged 13–17 years, with incidence rate ratios (IRRs) of 1.04 and 1.03 for ages 13–15 and 16–17, respectively [10]. Similarly, registry-based data from Norway demonstrated a major increase in pediatric ACL reconstructions between 2005 and 2021 [11]. Broader reviews confirm that ACL reconstruction techniques, graft preferences, and adjunctive procedures have undergone substantial changes in recent years, reflecting the global trend toward individualized, outcome-driven surgical practice [12,13].

On the other hand, recent years have also brought a critical reassessment of certain arthroscopic procedures, particularly in older patients with early degenerative knee pathology. Randomized controlled trials and national registry studies suggest that arthroscopic meniscectomy offers limited benefit for patients with early osteoarthritis compared to conservative treatment. This has led to a decline in routine arthroscopies for degenerative conditions in several high-income countries. In the United States, for example, registry data from 2010 to 2020 demonstrated a compound annual decrease of approximately 4% in the number of partial meniscectomies, accompanied by a significant increase in meniscus-preserving procedures such as meniscal repair [14]. A similar shift toward meniscus preservation has also been observed in Europe [15].

At the same time, the upward trajectory of ACL reconstructions that characterized earlier decades may now be reaching a plateau, or even showing mild pre-pandemic decline in some regions [16]. Globally, the COVID-19 pandemic caused a sharp drop in elective orthopedic procedures, including ACL reconstructions [16,17]. Following the easing of restrictions, however, a rapid rebound in surgical activity was observed, with most institutions striving to compensate for the backlog of deferred cases. Globally, current estimates suggest that ACL reconstruction volumes are once again increasing and may soon reach or even surpass pre-pandemic projections [16].

In Romania, arthroscopic knee surgery has been practiced in major centers for over two decades. Nevertheless, no nationwide epidemiological analyses have yet been published regarding the incidence or distribution of ACL reconstructions and other arthroscopic procedures. The absence of such data limits the ability to make meaningful comparisons with other countries and complicates strategic planning for healthcare resources, including workforce needs, infrastructure, and the development of regional referral centers. The present study aims to provide an overview of surgical trends in the management of ACL ruptures in Romania. We analyze national-level data from 2017 to 2023 concerning relevant knee arthroscopic procedures (ACL reconstructions, meniscectomies, synovectomies), stratified by year, sex, age group, geographic region, and hospital type (public vs. private). Furthermore, we interpret these findings in the context of international literature, highlighting similarities or discrepancies, and assessing the role of the COVID-19 pandemic and other factors in shaping these surgical trends.

## 2. Materials and Methods

### 2.1. Study Design and Data Source

We conducted a descriptive cross-sectional study using secondary analysis of national hospital administrative data. The source of information was the database compiled by the National Health Insurance House (CNAS), which aggregates reports from all Romanian hospitals (public and private) contracted under Ministry of Health Order 1782/2006. From this database, we extracted all records between 2017 and 2023 corresponding to arthroscopic knee procedures associated with anterior cruciate ligament (ACL) injury management. Procedures were identified using the Nordic classification of surgical procedures (NCSP) (Table 1) [18].

Extracted variables included year, hospital, county, sector (public/private), patient sex, and predefined age group. The study population consisted of all patients undergoing at least one of the above procedures during the study period, as reported in the CNAS system. The CNAS DRG database includes all inpatient procedures performed in public hospitals and in private facilities contracted with the national health insurance system. However, interventions carried out exclusively in out-of-pocket private settings, such as non-contracted clinics, are not captured. As such, the dataset reflects the vast majority of reimbursed hospital-based procedures nationwide but does not fully account for cases treated in the purely private sector. While no formal national estimate of this gap is available, the impact is likely moderate. Nevertheless, the exclusion of non-reimbursed cases may lead to a systematic underestimation of ACL reconstruction incidence, particularly among younger, privately insured patients who may opt for self-paid surgery to avoid waiting lists. Reported figures should thus be interpreted as conservative estimates of national surgical volumes.

### 2.2. Population Denominators and Incidence Calculation

Incidence rates were calculated as:

$$Incidence\;rate=\frac{Number\;of\;cases}{Population}\times 100,000$$

Annual population data (2017–2023), stratified by sex and age, were obtained from Eurostat [19] and the Romanian National Institute of Statistics (INSSE) [20]. For county-level analyses, population denominators were taken from the 2021 Romanian Population and Housing Census [21].

To enable valid between-country comparisons, we also computed age- and sex-standardized incidence rates for arthroscopic ACL reconstructions (O15303) using the European Standard Population (ESP 2013) as reference [22]. Standardized rates were calculated by direct standardization, applying age-specific incidence values to corresponding ESP weights across 16 five-year age groups (5–9 to 80–84 years).

The standardized incidence rate was derived using the formula:

$$\text{Standardized incidence rate}=\;\frac{\sum (I_{a}\times W_{a})}{\sum W_{a}}$$

where *I_a_* represents the crude incidence rate for age group a (per 100,000), and *W_a_* denotes the corresponding ESP weight.

### 2.3. Data Processing and Analysis

Data were consolidated and analyzed using Microsoft Excel and JASP (v0.19.3). We computed absolute numbers, proportions, and incidence rates (per 100,000 population). Group comparisons were performed using chi-square tests for categorical variables. A *p*-value < 0.05 was considered statistically significant (two-tailed). Results are presented as absolute numbers, proportions, incidence rates, and, where appropriate, 95% confidence intervals. To examine temporal trends and assess the potential impact of the COVID-19 pandemic on arthroscopic procedure incidence, we employed segmented linear regression using annual rates per 100,000 inhabitants. The model was fitted separately for the pre-pandemic (2017–2019) and post-pandemic (2021–2023) periods, excluding 2020 due to widespread suspension of elective surgeries. Risk ratios (RR) with 95% confidence intervals were computed for ACL reconstructions by 5-year age bands using sex-stratified counts. A continuity correction was applied where needed.

**Figure 1:**
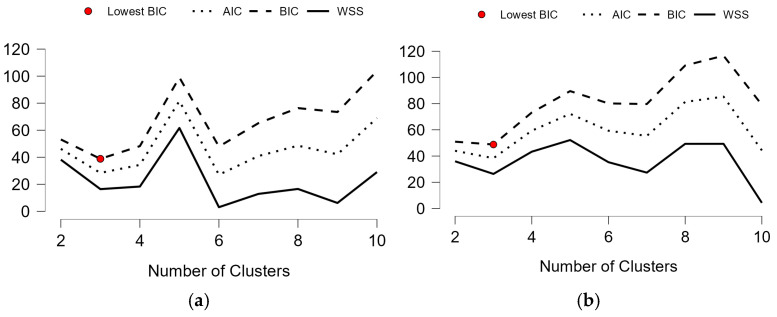
Cluster model selection based on BIC for (**a**) all arthroscopic procedures; (**b**) ACL reconstructions.

To investigate regional disparities, we performed fuzzy c-means clustering. The optimal number of clusters was determined based on Bayesian Information Criterion (BIC) minimization. In both models, a three-cluster solution yielded the best fit (Figure 1a,b). Input variables included the total number of procedures and the cumulative incidence per 100,000 inhabitants.

To assess whether the post-pandemic recovery differed between sectors, we performed a stratified trend analysis of ACL reconstructions using ANCOVA, with sector (public vs. private) as a fixed factor, year as a covariate, and an interaction term. The outcome was modeled as the annual percentage of procedures performed in each sector, relative to total national ACL volume.

## 3. Results

### 3.1. General Overview and Temporal Trend

During the study period, a total of 76,804 knee arthroscopic procedures were recorded in Romania. Among these, 29,979 cases (47.5%) were arthroscopic meniscectomies (O13404), making this the most frequently performed procedure. Arthroscopic knee reconstructions (O15301 and O15303 combined) accounted for 26,888 cases (42.6%), while arthroscopic synovectomies (O13405) represented 15,501 cases (24.5%). The least common procedures were arthroscopic excisions of meniscal or synovial plica lesions (O13401), with 4436 cases (7.0%).

As shown in Table 2, arthroscopic meniscectomy (O13404) was consistently the most frequent intervention, with an overall upward trend from 3905 cases in 2017 to 5542 in 2023, corresponding to an incidence increase from 19.9 to 29.1 per 100,000 inhabitants.

By contrast, arthroscopic excisions of meniscal or plica lesions (O13401) declined gradually, from 784 cases (3.99/100 k) in 2017 to 666 cases (3.50/100 k) in 2023. Arthroscopic synovectomy (O13405) remained relatively stable, with 2321–2543 annual cases (11.8–13.4/100 k), except for a marked decline in 2020 (1449 cases; 7.5/100 k) during the COVID-19 pandemic. Arthroscopic knee reconstructions coded as nonspecific (O15301) decreased from 2817 (14.3/100 k) in 2017 to 2184 (11.5/100 k) in 2023, likely reflecting a coding shift toward the specific ACL reconstruction code (O15303).

**Figure 2:**
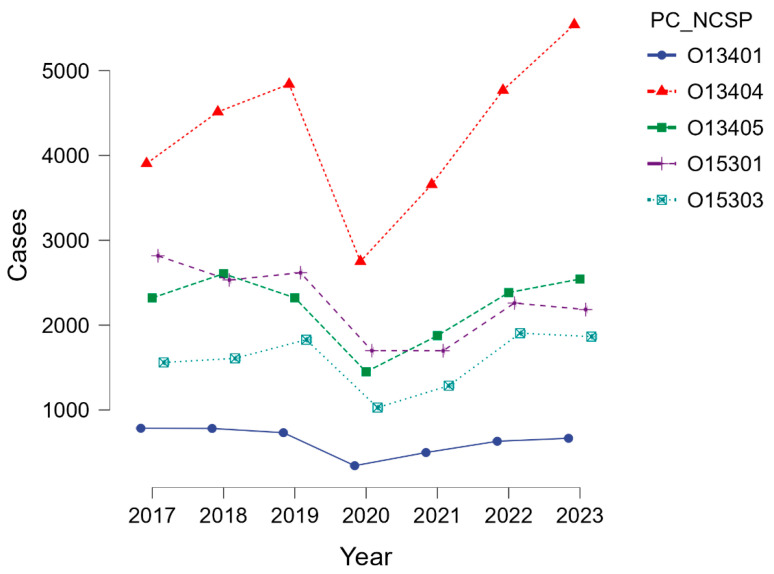
Annual trends in knee arthroscopic procedures. PC_NCSP = procedure code_Nordic classification of surgical procedures.

Arthroscopic ACL reconstructions (O15303) demonstrated a distinct pattern (Figure 2). Annual case numbers increased from 1560 (7.94/100 k) in 2017 to 1828 (9.42/100 k) in 2019, before dropping sharply to 1028 (5.32/100 k) in 2020, coinciding with the nationwide suspension of elective surgery. Recovery was evident in 2021 (1286 cases; 6.70/100 k), followed by a peak in 2022 (1905; 10.00/100 k), which slightly exceeded pre-pandemic levels. Incidence in 2023 remained high (1865; 9.79/100 k).

To evaluate temporal changes in incidence trends before and after the pandemic, a segmented linear regression model was fitted excluding the year 2020. While the estimated slope increased from 0.74 (2017–2019) to 1.55 (2021–2023) cases per 100,000 per year, the interaction term was not statistically significant (*p* = 0.522), likely due to the limited number of time points.

### 3.2. Sex Distribution

As summarized in Table 3, most knee arthroscopic procedures in Romania were performed in nearly equal proportions between men and women over the 2017–2023 period. Arthroscopic meniscectomy (O13404) showed an almost balanced distribution (15,239 male cases, 23.09/100 k; 14,740 female cases, 21.30/100 k). Similar patterns were observed for arthroscopic synovectomy (O13405: 51% male vs. 49% female) and nonspecific knee reconstructions (O15301: 11.93/100 k in men vs. 11.47/100 k in women). Arthroscopic excisions of meniscal or plica lesions (O13401) also demonstrated a symmetric distribution (2198 vs. 2238 cases).

**Figure 3:**
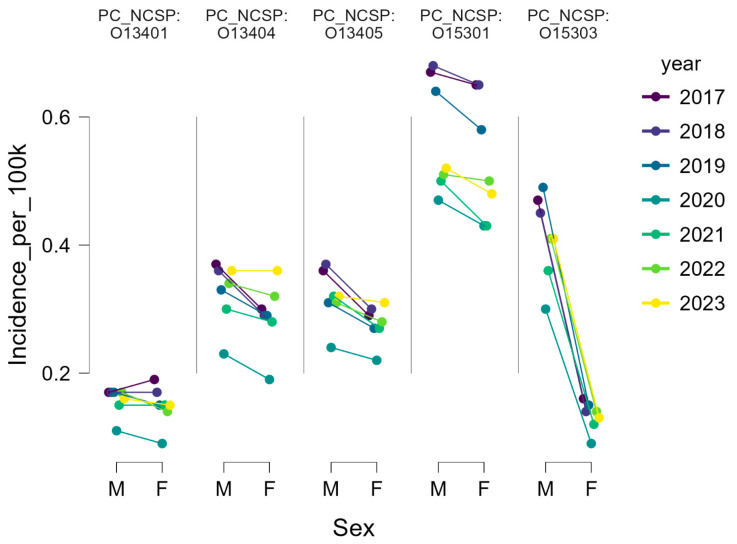
Annual distribution of knee arthroscopic procedures by sex, Romania 2017–2023.

By contrast, arthroscopic ACL reconstructions (O15303) displayed a marked male predominance, with 8226 male cases (12.46/100 k) compared with 2854 female cases (4.12/100 k), corresponding to a ratio of approximately 3:1. Men accounted for the majority of ACL reconstructions throughout the study period (Figure 3). The annual male-to-female ratio remained relatively constant at approximately 3:1, with men representing between 72% and 76% of cases. For example, in 2019 there were 1374 reconstructions in men (14.46/100,000) compared with 454 in women (4.58/100,000). During the pandemic year 2020, both groups experienced a marked decline, with incidence dropping to 8.16/100,000 in men and 2.59/100,000 in women, but the sex ratio remained unchanged.

Following 2020, both sexes demonstrated recovery, with the highest annual numbers recorded in 2022: 1425 male cases (15.28/100,000) and 480 female cases (4.81/100,000). This was slightly above pre-pandemic levels, indicating not only the rescheduling of postponed cases but also the persistence of an upward trend in overall surgical demand.

Chi-square analysis confirmed a significantly male-dominant distribution in ACL reconstructions (O15303; *p* < 0.001). Minor but statistically significant sex differences were also observed in meniscectomies (O13404; *p* = 0.004) and synovectomies (O13405; *p* = 0.007), whereas procedures O13401 and O15301 displayed no significant deviation from a balanced male-to-female ratio

### 3.3. Age Distribution

The age distribution of knee arthroscopic procedures varied according to intervention type. Arthroscopic meniscectomy (O13404) demonstrated the highest volumes in middle-aged adults, peaking in the 50–54-year group (4824 cases; 34.85/100 k) and remaining frequent up to 70 years of age. This age distribution suggests that meniscectomy is predominantly performed in adults over 40, with sustained demand in later decades of life.

Arthroscopic synovectomy (O13405) presented a more uniform age distribution, with most procedures performed between 30 and 60 years (5–8/100 k), consistent with the typical onset age of chronic synovitides. Nonspecific reconstructions (O15301) and arthroscopic excisions of meniscal or plica lesions (O13401) showed smaller volumes, mostly concentrated in young and middle-aged adults (Table 4).

**Figure 4:**
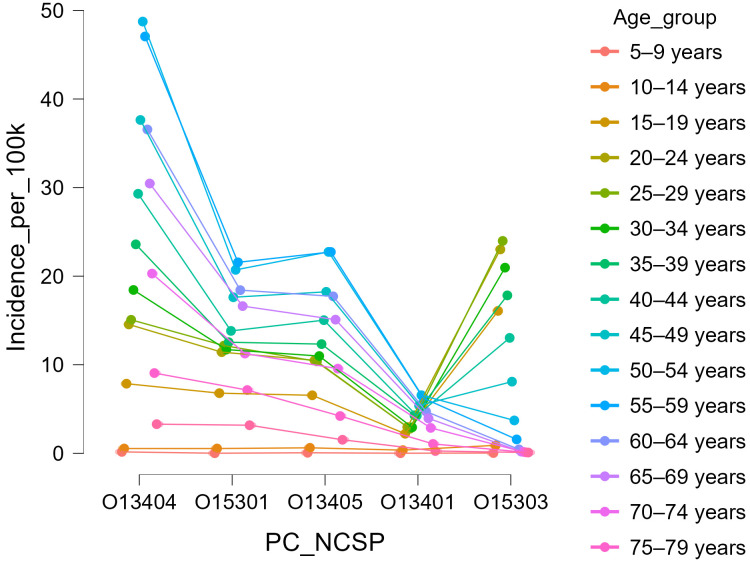
Cumulative age-specific incidence (per 100,000 inhabitants) of knee arthroscopic procedures.

Figure 4 illustrates the cumulative age-specific incidence (per 100,000 inhabitants) for each arthroscopic procedure across the 2017–2023 period. Distinct age profiles were observed: arthroscopic meniscectomy (O13404) peaked in middle-aged adults (50–54 years), synovectomy (O13405) showed a broad mid-life distribution, whereas ACL reconstruction (O15303) predominated among young adults aged 15–34 years.

**Figure 5:**
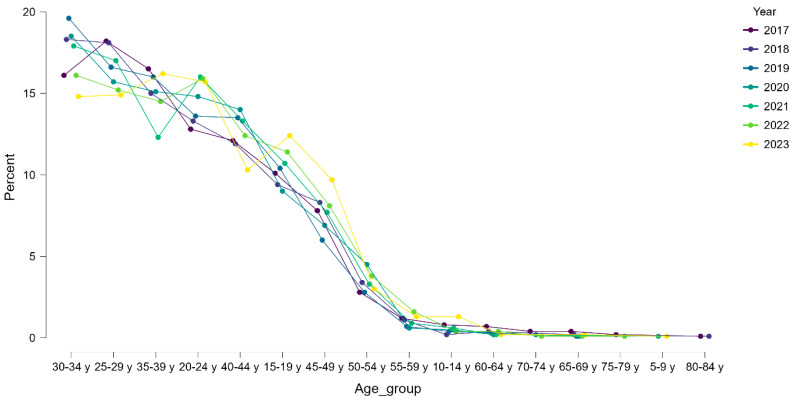
Relative annual age distribution of arthroscopic ACL reconstructions (O15303) in Romania, 2017–2023.

By contrast, arthroscopic ACL reconstructions (O15303) showed a sharply different age pattern. Over 80% of ACL reconstructions were performed in patients younger than 40 years, with a cumulative peak in the 25–34-year groups, where incidence exceeded 24/100 k. Figure 5 displays the relative annual age distribution of ACL reconstructions, confirming that this profile remained stable throughout the study period. Even in 2020, when surgical activity decreased nationwide, the age structure of ACL cases was preserved, indicating a stable demographic profile of surgical demand.

To further explore these sex differences across age, we performed a stratified risk ratio analysis for ACL reconstruction (O15303) by 5-year age bands (Table 5). The analysis confirmed a consistently elevated risk among males, particularly between 20 and 39 years of age. The highest relative risks were observed in the 25–29 and 30–34-year groups, with RR = 4.18 (95% CI: 3.61–4.84) and RR = 4.13 (95% CI: 3.57–4.78), respectively. In all age groups under 40, male patients were over 2.5 times more likely to undergo ACL reconstruction compared to females. These results reinforce the demographic profile of ACL surgery as predominantly targeting young, physically active men and underscore the importance of sex-sensitive prevention and rehabilitation strategies.

To complement the raw and stratified comparisons, we calculated age- and sex-standardized incidence rates for ACL reconstruction using the 2013 European Standard Population (ESP). The standardized incidence for the total population was 9.93 per 100,000, with males exhibiting a significantly higher rate (13.65/100,000) than females (6.19/100,000). The male predominance is evident across nearly all age groups, particularly between 20–39 years, where incidence among males consistently exceeded 24 per 100,000 (Table 6).

### 3.4. Regional Distribution

The geographical distribution of knee arthroscopic procedures in Romania during 2017–2023 was highly uneven. When incidence was adjusted to population size (using 2021 census data as denominator), the highest cumulative rates were recorded in Bihor (1557.4/100,000), Timiș (1266.8/100,000), Mureș (1021.0/100,000), and Bucharest (1128.9/100,000), all major regional referral centers with established arthroscopic services and orthopedic training programs.

**Figure 6:**
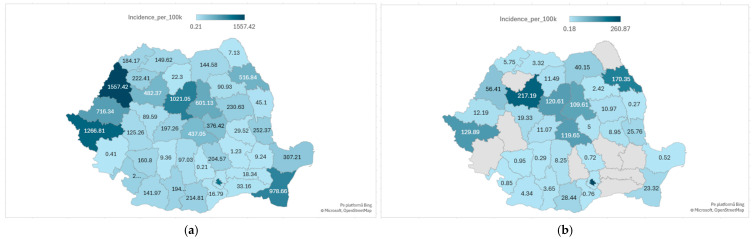
Regional distribution of knee arthroscopic procedures in Romania, 2017–2023. (**a**), cumulative incidence (per 100,000 inhabitants) of all knees arthroscopic procedures; (**b**), cumulative incidence of arthroscopic ACL reconstructions (O15303).

By contrast, several counties reported extremely low surgical activity, corresponding to negligible incidence values, Dâmbovița (0.2/100,000; 1 case), Caraș-Severin (0.4/100,000; 1 case), Buzău (1.2/100,000; 5 cases), Botoșani (7.1/100,000; 28 cases), and Brăila (9.2/100,000; 26 cases). These striking discrepancies, illustrated in Figure 6a, reflect the lack of local arthroscopic expertise and highlight marked regional inequalities in access to surgical care.

The geographical distribution of ACL reconstructions (O15303) was even more centralized than that of overall knee arthroscopic procedures. Bucharest alone accounted for 4479 reconstructions (260.9/100,000; approximately 40% of all national cases), followed by Cluj (1475; 217.2/100,000), Iași (1296; 170.4/100,000), Timiș (845; 129.9/100,000), and Brașov (654; 119.7/100,000). Together, these five counties performed more than two-thirds of all ACL reconstructions nationwide.

In contrast, several peripheral regions reported very limited surgical volumes, with many counties recording fewer than 15 reconstructions per year on average. This pattern, depicted in Figure 6b, underlines the dominance of a few university-based centers compared with sparse or absent activity in large parts of the country.

**Figure 7:**
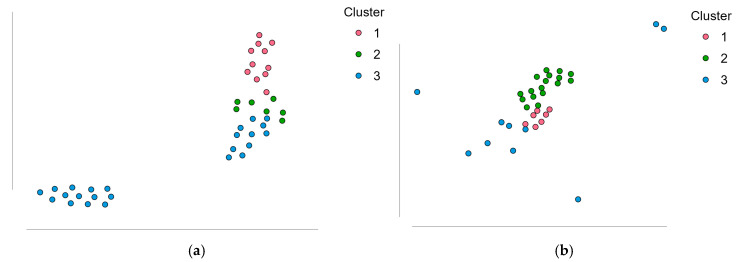
Cluster analysis of county-level surgical activity. (**a**). clustering of all arthroscopic procedures based on incidence and total case number; (**b**). clustering of ACL reconstructions (O15303), including counties with zero reported cases.

To explore these patterns further, we applied a cluster analysis based on incidence and total case volume. For all arthroscopic procedures, three distinct county profiles emerged (Figure 7a). Cluster 1 (*n* = 11) encompassed high-access regions with above-average incidence and case volumes, primarily major academic centers such as Bucharest, Timiș, Cluj, Bihor, and Mureș. Cluster 2 (*n* = 7) included intermediate-access counties, such as Galați, Bacău, Tulcea, and Suceava, with values close to the national mean. Cluster 3 (*n* = 24) captured most counties, characterized by consistently low surgical activity, including Mehedinți, Olt, Ialomița, and Satu Mare. These profiles suggest that, although general arthroscopic procedures are performed across most regions, access remains highly asymmetric.

For ACL reconstructions, clustering revealed a more polarized landscape (Figure 7b), but with a distinct pattern. Cluster 3 (*n* = 10) grouped the few counties with consistently high ACL volumes and incidence, such as Bucharest, Cluj, Iași, Timiș, and Brașov, representing the dominant referral hubs. In contrast, Clusters 1 and 2 (*n* = 32) comprised counties with either marginal surgical activity or no reported ACL procedures at all, despite some having performed other arthroscopic interventions (e.g., Hunedoara, Brăila, Caraș-Severin, Sălaj).

**Figure 8:**
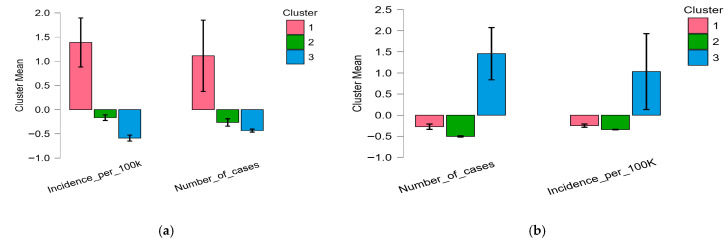

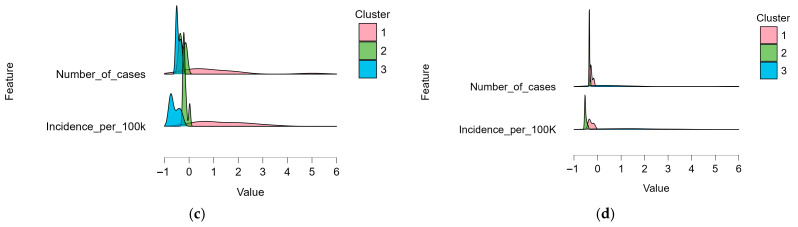
Cluster analysis of regional arthroscopic surgical activity in Romania (2017–2023): (**a**). cluster mean plot for all arthroscopic procedures; (**b**). feature density plot for all arthroscopic procedures; (**c**). cluster mean plot for ACL reconstructions (O15303); (**d**). feature density plot for ACL reconstructions.

These results were further validated through cluster mean plots and distribution density graphs (Figure 8), which confirmed the high concentration of procedures in a limited number of referral hubs, and the widespread underutilization or unavailability of arthroscopic services across much of the country.

### 3.5. Public vs. Private Sector Distribution

As summarized in Table 7, most arthroscopic knee procedures in Romania during 2017–2023 were performed in public hospitals, which accounted for nearly 90% of all cases. The private sector contributed only about 11% overall, but its role varied considerably across procedure types.

For arthroscopic meniscectomies (O13404) and synovectomies (O13405), private hospitals played only a minor role (≈8% of cases), reflecting the fact that these interventions are widely available in public institutions and are often performed in patients with degenerative or chronic conditions, who typically remain in the public system. Marginal excisions of meniscal plica (O13401) were almost exclusively carried out in public hospitals (>98%).

By contrast, the private sector had a substantially higher share in ligament reconstructions. For non-specified arthroscopic reconstructions (O15301), approximately 20% of cases were performed in private settings, while for ACL reconstructions (O15303), the private sector accounted for nearly 18% of procedures.

**Figure 9:**
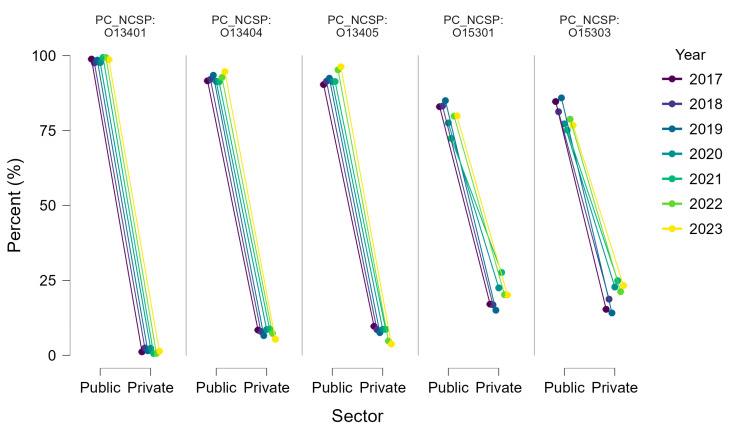
Distribution of arthroscopic knee procedures between public and private hospitals in Romania, 2017–2023.

The relative contribution of private hospitals increased after 2020, particularly for ligament reconstructions, consistent with the expansion of private orthopedic centers and the impact of the COVID-19 pandemic on public-sector elective surgery capacity. The distribution of procedures between public and private hospitals is illustrated in Figure 9, which shows that while the public sector consistently dominates all categories, the private sector has a disproportionately higher share in ligament reconstructions compared to meniscal or synovial procedures.

**Figure 10:**
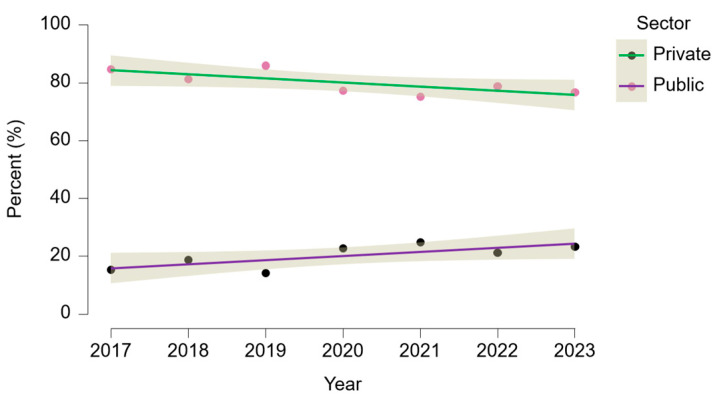
Annual trend in ACL reconstructions (O15303) by hospital sector in Romania, 2017–2023. Lines represent linear regression fits by sector, with 95% confidence bands.

This observation was supported by inferential statistics: an ANCOVA on the annual percentage of ACL reconstructions confirmed a significant main effect of sector (F(1,10) = 12.49, *p* = 0.005) and a significant interaction between sector and year (F(1,10) = 12.23, *p* = 0.006), indicating that trends in procedure share evolved differently across the two sectors. No significant linear effect was found for year alone (*p* = 1.000), suggesting that the shift was not gradual over time but was largely driven by post-2020 recovery and private sector expansion, as can be seen in Figure 10.

## 4. Discussion

Arthroscopy represents one of the most transformative advances in modern orthopedics, offering precise, minimally invasive management of joint pathology with shorter recovery and improved outcomes [23]. In this context, as a direct continuation of our previous nationwide analysis of hospitalized acute ACL ruptures in Romania [24], the present study provides the first nationwide evaluation of arthroscopic knee surgery in Romania, using comprehensive health insurance data from 2017–2023. The analysis reveals a steady pre-pandemic increase in anterior cruciate ligament (ACL) reconstructions, a sharp but transient decline during the pandemic, and a rapid subsequent recovery, patterns that closely mirror global trends in elective arthroscopic activity.

Between 2017 and 2019, the number of ACL reconstructions increased from 1560 (7.94 per 100,000 inhabitants) to 1828 (9.42/100 k), followed by a sharp decline in 2020 (1028; 5.32/100 k). Recovery occurred quickly, reaching 1286 (6.70/100 k) in 2021 and peaking at 1905 (10.00/100 k) in 2022 before stabilizing at 1865 (9.79/100 k) in 2023. Overall, this represents a 19.6% net increase between 2017 and 2023, excluding the pandemic year. Standardized to the 2013 European population structure, the national incidence of ACL reconstruction was 9.93 per 100,000 inhabitants, with markedly higher rates among males (13.65/100 k) compared to females (6.19/100 k). This pattern reflects the sex-specific burden observed in the crude data and confirms the demographic asymmetry in surgical uptake. While this trajectory broadly mirrors international trends, Romania’s incidence remains considerably lower than in countries such as Australia (77/100 k) [25], Canada (51/100 k) [26], and the Nordic countries [27]. However, direct comparison is limited by differences in data sources, population coverage, and reporting practices. As summarized in Table 8, our estimates are based on inpatient DRG data reported to the national insurer (CNAS) and likely under-capture procedures performed in non-contracted private facilities or outpatient-only settings. In contrast, many high-income countries include outpatient and day-surgery procedures and often rely on specialized registries with broader coverage. These methodological differences should be considered when interpreting cross-national incidence gaps.

The 44% reduction in 2020 coincides with global observations of pandemic-related surgical interruption, where reductions of 50–80% in elective orthopedic procedures were reported [28,29]. The rapid rebound in 2021–2022 suggests system resilience and effective recovery of postponed cases. It may also reflect a temporary increase in injury incidence as physical activity resumed after prolonged restrictions [29]. The Romanian data thus illustrate both the vulnerability and the adaptability of national surgical systems to external shocks.

Demographically, ACL reconstruction remains predominantly a procedure of young men (74.2% of cases; 8226 males vs. 2854 females), a proportion consistent with European averages [30,31,32]. Over 80% of ACL reconstructions were performed in patients under 40 years, peaking in the 30–34-year group (1907; 20.96/100 k). Risk ratio analysis confirmed a markedly increased likelihood of surgery among men, particularly in the 20–39-year range, with the highest male-to-female risk observed in the 25–29 and 30–34 age groups (RR = 4.18 and 4.13, respectively). These data reinforce the profile of ACL injury as one disproportionately affecting active young males.

One plausible explanatory factor for this sex disparity may be differential exposure to high-risk sports. Lower rates of organized sport participation among adolescent girls, especially in pivoting disciplines such as football, handball, and basketball, may contribute to the reduced ACL injury burden and surgical demand in this population. This hypothesis is supported by international data linking sport specialization and high training volume to increased ACL injury risk, particularly among youth athletes [33]. Emerging evidence also points to important sex- and gender-related differences in psychological readiness for return to sport, functional outcomes, and rehabilitation adherence, with males often reporting higher confidence and activity levels post-reconstruction [34]. These differences may contribute to observed disparities in access and outcomes, highlighting the importance of individualized rehabilitation strategies and increased awareness among clinicians regarding sex-sensitive care. Incorporating gender-specific analysis into national registries and outcome assessments would support a more inclusive and equitable orthopedic practice. In addition, socioeconomic frictions such as out-of-pocket payments, travel distance, or long waiting times in public hospitals [35] may further deter women, particularly younger, early-career patients, from seeking surgical care. However, the current dataset does not include variables such as income, insurance coverage, or geographic location, limiting our ability to formally test these mechanisms. Future linkage between hospital and socioeconomic data would help clarify the access dynamics underlying this disparity.

By contrast, arthroscopic meniscectomy (O13404) displayed a distinct age profile, with the highest incidence in middle-aged adults (50–54 years: 4824 cases; 48.74/100 k), persisting into older age groups. While this pattern aligns with age brackets commonly associated with degenerative meniscal pathology, the absence of diagnostic codes in our dataset precludes direct attribution. Nonetheless, the observed age profile is consistent with patterns reported in studies of meniscectomy use for degenerative conditions [36,37]. International guidelines, have increasingly favored conservative approaches for patients with degenerative meniscal tears, citing comparable functional outcomes, lower cost, and fewer complications compared to surgery [37,38,39,40]. In parallel, many health systems have adopted a “meniscus-preserving” philosophy, with a notable increase in repair procedures, especially among younger patients with traumatic tears [41,42].

By contrast, Romania continues to report high meniscectomy rates and limited use of meniscal repair, though national data on repair volumes are currently lacking. This imbalance may reflect systemic constraints, such as limited physiotherapy access, variability in surgical training, and financial incentives favoring excision. A structured national audit comparing meniscectomy and repair volumes, alongside outcome monitoring, would be instrumental in assessing the alignment of Romanian practice with current international standards and identifying opportunities for clinical and policy reform.

Our age-specific analyses suggest a distinct etiological profile for different arthroscopic procedures. Meniscectomy incidence peaked in middle-aged and older adults, with the highest rates recorded in the 50–54-year group (4824 cases; 48.74/100 k), supporting the predominance of degenerative meniscal pathology in this cohort. In contrast, ACL reconstruction was overwhelmingly performed in younger adults, particularly in the 20–39-year range, aligning with the typical age of traumatic, sports-related injuries [34].

While procedural codes do not differentiate explicitly between traumatic and degenerative lesions, the demographic stratification provides indirect etiological insight. Future registry-based analyses could strengthen these inferences by integrating diagnostic codes, injury mechanisms, and imaging data. This would enable more accurate mapping of surgical indications and better alignment with international guidelines recommending conservative management for degenerative meniscal tears.

A striking feature of the current data is the strong regional centralization of arthroscopic surgery. Five university-based centers, Bucharest (4479 ACL reconstructions; 260.9/100 k), Cluj (1475; 217.2/100 k), Iași (1296; 170.4/100 k), Timiș (845; 129.9/100 k), and Brașov (654; 119.7/100 k), accounted for over two-thirds of national ACL reconstructions, reflecting a concentration of expertise, equipment, and patient referral networks. In contrast, several counties reported negligible surgical activity, illustrating major geographic inequities. These patterns resemble those observed in other Central and Eastern European countries [43] and highlight the need to decentralize arthroscopic services through capacity building, workforce training, and structured referral systems. These results were further substantiated through clustering analyses, which stratified Romanian counties into three distinct access profiles for both overall arthroscopy and isolated ACL reconstructions. The clustering model based on all knee arthroscopies delineated a small group of high-access counties with advanced surgical activity, a moderate-access group with near-average values, and a broad low-access cluster encompassing most regions. The ACL-specific clustering showed an even more polarized distribution, isolating a handful of high-volume centers while grouping 32 counties, including several with some arthroscopic capacity, into low- or zero-access clusters. These findings emphasize that ACL surgery is not merely centralized, but structurally restricted to a few dominant hubs.

Such disparities likely reflect broader systemic limitations in the Romanian healthcare system. Specialized orthopedic services remain concentrated in university-affiliated hospitals, benefiting from trained personnel, infrastructure, and stable referral networks. In contrast, peripheral counties often lack not only surgical capacity but also imaging and rehabilitation services required for complex procedures. Additional factors, including uneven population distribution, patient mobility, and socioeconomic constraints, further deepen access gaps. Prior analyses have described similar structural barriers to equitable healthcare delivery in Romania, underscoring the interplay between geographic, economic, and institutional determinants [44,45,46].

Addressing these inequalities requires targeted investment in regional surgical capacity, expanded orthopedic training programs, and the development of coordinated referral systems. Establishing a national ACL registry would support quality monitoring, enable evidence-based planning, and promote adherence to treatment standards [47,48].

Beyond structural constraints, variation in regional procedure rates may also reflect differences in population activity patterns and exposure to injury risk. Counties with higher participation in organized sports, physically demanding occupations, or urban youth populations may exhibit increased incidence of traumatic knee injuries, particularly ACL ruptures. These epidemiological factors are not captured in administrative datasets but likely contribute to the clustering profiles observed. Additionally, life-course considerations, such as pregnancy, childcare responsibilities, and employment patterns, may influence the timing or likelihood of surgical intervention in women. Recent literature highlights how gendered sport environments, marked by structural inequities, gender norms, and devaluation of women’s sports, may further constrain injury management pathways for female athletes, particularly in the absence of tailored support structures [34,49]. These factors could partially explain the consistently lower proportion of ACL reconstructions among females, particularly in their twenties, and warrant further exploration through patient-reported data. Furthermore, the national procedural landscape remains dominated by reparative and reconstructive interventions, such as meniscectomy and ACL reconstruction, with limited uptake of cartilage-preserving or regenerative techniques. This may reflect both resource limitations and prevailing surgical paradigms. Future integration of diagnostic codes, injury mechanisms, and treatment strategies would clarify these trends and support more nuanced planning.

From a health system perspective, 89.1% of all arthroscopic procedures were performed in public hospitals, while 10.9% occurred in private facilities. However, for ACL reconstructions, the private sector accounted for 17.8% of procedures, reflecting growing reliance on private services. The private sector’s expansion after 2020 aligns with a broader European trend of increased private involvement in elective orthopedic care [50]. This tendency was also reflected in our data, which revealed a disproportionate post-2020 rise in private-sector ACL surgeries. Statistical analysis confirmed that this growth differed significantly from trends in the public sector, underscoring the role of private providers in absorbing unmet demand during and after the pandemic. While beneficial for reducing waiting times, this shift raises questions about financial equity and the long-term sustainability of dual-sector delivery models [51]. However, national data on waiting times and patient redirection between sectors remain limited. Furthermore, the CNAS database captures only private hospitals contracted with the national insurance system, excluding surgeries paid fully out-of-pocket in purely private centers. As such, the true scale and demographic profile of private-sector ACL reconstructions is likely underestimated. A structured analysis of intersectoral patient flow and time-to-treatment metrics would offer valuable insight into access dynamics and help shape equitable surgical planning.

The broader context of these findings reflects the ongoing modernization of arthroscopy in Romania. The steady rise in procedure volumes, coupled with improved data precision and expanding private participation, indicates increasing integration of contemporary minimally invasive techniques. As global arthroscopy moves toward biologically enhanced repair, 3D visualization, and patient-specific rehabilitation, population-level data such as ours provide valuable insight into adoption patterns and unmet needs. Future work should extend this foundation by linking national procedural data with patient-reported outcomes, functional recovery metrics, and cost-effectiveness analyses.

One actionable step to improve national oversight and standardization of ACL reconstruction in Romania would be the implementation of a national ACL registry. Modeled after successful examples in Scandinavia and Western Europe, such a registry should ideally collect standardized perioperative and postoperative data across both public and private hospitals. Core variables would include patient demographics, graft type, surgical technique, intraoperative findings, postoperative complications, and functional outcomes assessed at predefined intervals [47,48]. In addition to supporting longitudinal analysis of recurrence, failure rates, and surgical revisions, a well-structured registry would also enable benchmarking against international guidelines and facilitate integration into multicenter European collaborations focused on evidence-based practice and surgical innovation. Over time, such infrastructure could also serve as a platform for cost-effectiveness analyses, quality assurance initiatives, and the deployment of digital tools for patient-reported outcomes. Successful models from Scandinavia, including the Norwegian, Danish, and Swedish ACL registries, have demonstrated the feasibility and clinical utility of such systems in improving care quality, monitoring complications, and supporting international benchmarking [52,53,54].

Our research has several limitations. It relies on administrative data, which lack detailed clinical variables such as injury mechanism, graft choice, severity of concomitant meniscal or chondral lesions, postoperative complications, and functional outcomes. As a result, it was not possible to stratify patients by case complexity or to assess adherence to clinical recommendations regarding graft selection, meniscal repair, or rehabilitation protocols. The distinction between traumatic and degenerative lesions could not be made, nor could long-term outcomes be evaluated. While coding systems are standardized, occasional misclassification or reporting inconsistencies may occur, although their effect is likely small given the large dataset and internal consistency. The absence of diagnostic linkage (e.g., ICD-10 codes) also limited our ability to reclassify non-specific ligament reconstructions (O15301) and determine whether the observed decline in their use reflects true changes in clinical practice or improved procedural coding. Procedures performed entirely outside the public insurance system were not captured but are presumed to represent a minor fraction. Population estimates were based on census and official projections, which may not fully reflect temporary migration flows or regional demographic shifts. Waiting times and patient flow between sectors were not available, limiting the interpretation of access disparities between public and private providers. The study also excluded several arthroscopic procedures relevant to osteoarthritis management, such as chondroplasty, loose body removal, cartilage repair, or lavage, due to inconsistent coding in the national database. Although increasingly used in joint preservation strategies, these interventions could not be reliably identified for national-level analysis. The absence of a national ACL registry further limited our ability to assess complications, reinterventions, or long-term outcomes, and constrained comparisons with international benchmarks. Finally, although year-to-year comparisons were performed using descriptive and inferential statistics, we did not apply advanced modeling approaches such as mixed-effects regression or time-series analysis. These techniques may allow better quantification of trends and pandemic-related deviations but were not included due to the short post-pandemic observation period. Future research should aim to address these gaps by incorporating clinical registries, longitudinal follow-up, and predictive modeling strategies.

Taken together, the above presented results provide a quantitative framework for understanding how arthroscopic practice in Romania has evolved during a period of technological and systemic change. The trends observed align with the global transition of arthroscopy from purely technical intervention to a multidisciplinary process integrating precision surgery, biological augmentation, and patient-centered rehabilitation [55,56,57]. Romania’s experience reflects both progresses, through growing procedural adoption and recovery after the pandemic, and persistent challenges in equity, guideline implementation, and infrastructure. Continued investment in education, registry development, and outcome tracking will be key to advancing arthroscopy and ensuring that these innovations translate into improved patient care.

## 5. Conclusions

This nationwide study provides the first comprehensive assessment of arthroscopic knee surgery in Romania, indicating sustained pre-pandemic growth in ACL reconstructions, a transient COVID-19-related decline, and full post-pandemic recovery exceeding previous activity levels. Persistent regional and demographic disparities underline the unequal distribution of surgical expertise and access to arthroscopic care. These disparities likely reflect not only infrastructural gaps but also differences in population activity levels, injury exposure, and referral patterns.

The findings emphasize the need to expand equitable access beyond university centers, standardize clinical practice in line with international guidelines—particularly regarding degenerative meniscal surgery—and promote evidence-based, meniscus-preserving strategies. Given the predominance of reparative and reconstructive procedures, future policy should also support the integration of cartilage-preserving and regenerative arthroscopic techniques. Establishing a national ACL registry and strengthening rehabilitation and injury-prevention programs would support data-driven policy, improve long-term functional outcomes, and align Romanian arthroscopic practice with contemporary international standards.

## Figures and Tables

**Table 1:** Arthroscopic knee procedures included in the study.

NCSP Code	Procedure	Description
O13401	Arthroscopic excision of meniscal rim or synovial plica	Limited partial meniscectomy or suprapatellar plica resection; often used for localized meniscal tears.
O13404	Arthroscopic meniscectomy	Subtotal or total meniscectomy performed arthroscopically.
O13405	Arthroscopic synovectomy	Resection of hypertrophic/inflamed synovium in chronic or proliferative synovitides.
O15301	Arthroscopic reconstruction of the knee, unspecified	Generic code for ligament reconstructions not otherwise specified (e.g., posterior cruciate ligament, multi-ligament reconstructions).
O15303	Arthroscopic anterior cruciate ligament (ACL) reconstruction with meniscal repair	Specific code for primary ACL reconstruction, usually combined with meniscal repair.

**Table 2:** Annual number of cases and incidence per 100,000 inhabitants of knee arthroscopic procedures.

Year	O13401 n (I/100 K)	O13404 n (I/100 K)	O13405 n (I/100 K)	O15301 n (I/100 K)	O15303 n (I/100 K)	Total n (I/100 K)
2017	784 (3.99)	3905 (19.88)	2321 (11.82)	2817 (14.34)	1560 (7.94)	11387 (57.97)
2018	782 (4.00)	4514 (23.11)	2606 (13.34)	2532 (12.96)	1608 (8.23)	12042 (61.65)
2019	732 (3.77)	4840 (24.93)	2322 (11.96)	2618 (13.48)	1828 (9.42)	12340 (63.56)
2020	343 (1.77)	2751 (14.23)	1449 (7.50)	1699 (8.79)	1028 (5.32)	7270 (37.61)
2021	498 (2.59)	3658 (19.05)	1876 (9.77)	1698 (8.84)	1286 (6.70)	9016 (46.95)
2022	631 (3.31)	4769 (25.04)	2384 (12.52)	2260 (11.87)	1905 (10.00)	11949 (62.75)
2023	666 (3.50)	5542 (29.08)	2543 (13.35)	2184 (11.46)	1865 (9.79)	12800 (67.18)

Procedure codes: O13401 = arthroscopic excision of meniscal or plica lesions; O13404 = arthroscopic meniscectomy; O13405 = arthroscopic synovectomy; O15301 = arthroscopic knee reconstruction (nonspecific); O15303 = arthroscopic ACL reconstruction.

**Table 3:** Cumulative number of cases and incidence per 100,000 inhabitants of knee arthroscopic procedures by sex.

Procedure Cod	Male n (I/100 K)	Female n (I/100 K)
O13401	2198 (3.33)	2238 (3.23)
O13404	15,239 (23.09)	14,740 (21.3)
O13405	7917 (11.99)	7584 (10.96)
O15301	7873 (11.93)	7935 (11.47)
O15303	8226 (12.46)	2854 (4.12)

**Table 4:** Cumulative number of cases and incidence per 100,000 inhabitants of knee arthroscopic procedures by age group.

Age Group	O13401	O13404	O13405	O15301	O15303
under 1 year	–	1 (nan)	–	–	–
1–4 years	–	–	–	1 (nan)	–
5–9 years	1 (0.01)	11 (0.16)	4 (0.06)	1 (0.01)	2 (0.03)
10–14 years	27 (0.36)	40 (0.54)	46 (0.62)	40 (0.54)	67 (0.9)
15–19 years	162 (2.21)	576 (7.86)	480 (6.55)	498 (6.79)	1179 (16.08)
20–24 years	208 (2.96)	1021 (14.55)	737 (10.5)	801 (11.42)	1615 (23.02)
25–29 years	211 (2.77)	1147 (15.07)	790 (10.38)	925 (12.16)	1825 (23.98)
30–34 years	267 (2.93)	1678 (18.44)	999 (10.98)	1065 (11.71)	1907 (20.96)
35–39 years	405 (4.29)	2227 (23.59)	1164 (12.33)	1184 (12.54)	1683 (17.83)
40–44 years	447 (4.25)	3080 (29.3)	1580 (15.03)	1453 (13.82)	1370 (13.03)
45–49 years	580 (5.4)	4044 (37.63)	1959 (18.23)	1894 (17.62)	870 (8.09)
50–54 years	649 (6.56)	4824 (48.74)	2251 (22.74)	2050 (20.71)	367 (3.71)
55–59 years	483 (6.2)	3663 (47.06)	1769 (22.73)	1678 (21.56)	122 (1.57)
60–64 years	422 (4.72)	3267 (36.56)	1583 (17.72)	1646 (18.42)	39 (0.44)
65–69 years	335 (3.95)	2582 (30.46)	1279 (15.09)	1409 (16.62)	16 (0.19)
70–74 years	180 (2.87)	1271 (20.29)	599 (9.56)	705 (11.26)	10 (0.16)
75–79 years	48 (1.06)	411 (9.06)	191 (4.21)	324 (7.14)	5 (0.11)
80–84 years	9 (0.26)	116 (3.29)	54 (1.53)	112 (3.17)	3 (0.09)
over 85	2 (nan)	20 (nan)	16 (nan)	22 (nan)	–

nan = incidence not calculated due to missing denominator data or small case numbers.

**Table 5:** Risk Ratios by Age Group.

Age Group	Male	Female	Risk Ratio	95% CI Lower	95% CI Upper
20–24	0	2	0.00	0.00	16.55
25–29	26	41	1.27	0.78	2.08
30–34	745	434	1.63	1.46	1.82
35–39	1293	322	3.54	3.14	3.99
40–44	1542	283	4.18	3.71	4.72
45–49	1598	309	4.13	3.67	4.65
50–54	1282	401	2.94	2.63	3.29
55–59	936	434	2.01	1.78	2.27
60–64	539	331	1.63	1.41	1.88
65–69	174	193	0.90	0.72	1.13
70–74	57	65	0.88	0.60	1.29
75–79	21	18	1.17	0.65	2.10
80–84	6	10	0.65	0.25	1.66
20–24	3	7	0.43	0.11	1.63
25–29	2	3	0.67	0.11	4.00
30–34	1	3	0.33	0.03	3.18

Estimates for age groups with fewer than 10 cases per sex are presented for completeness but should be interpreted with caution due to statistical instability.

**Table 6:** Age- and sex-standardized incidence rates of arthroscopic ACL reconstruction (O15303) using the 2013 European Standard Population (ESP).

Age Group	ESP Weight	Total Incidence (/100 k)	Male Incidence (/100 k)	Female Incidence (/100 k)
5–9	5500	0.03	0.01	0.04
10–14	5500	0.90	0.54	1.27
15–19	5500	16.08	20.54	11.51
20–24	6000	23.02	31.95	14.30
25–29	6000	23.98	34.13	13.99
30–34	6000	20.96	28.47	12.96
35–39	6000	17.83	24.21	11.22
40–44	6000	13.03	18.24	7.84
45–49	6000	8.09	11.31	5.06
50–54	6000	3.71	5.56	1.95
55–59	5000	1.57	2.35	0.76
60–64	5000	0.44	0.66	0.22
65–69	4000	0.19	0.28	0.09
70–74	3000	0.16	0.24	0.07
75–79	2000	0.11	0.17	0.04
80–84	1000	0.09	0.14	0.03

**Table 7:** Distribution of arthroscopic knee procedures by sector (public vs. private) in Romania, 2017–2023 (cumulative).

Procedure Code	Public n (%)	Private n (%)	Total Cases
O13401	3648 (98.5)	55 (1.5)	3703
O13404	28,577 (92.3)	2412 (7.7)	30,989
O13405	12,384 (92.0)	1070 (8.0)	13,454
O15301	9238 (80.1)	2294 (19.9)	11,532
O15303	8564 (82.2)	1856 (17.8)	10,420
Total	62,411 (89.1)	7687 (10.9)	70,098

**Table 8:** ACL reconstruction incidence reporting, comparability across countries, with denominator-General population.

Country	Source Type	Numerator	Setting	Comments
Romania	CNAS database	All reported ACL reconstructions	Inpatient only (DRG data)	Includes public & private contracted hospitals
Australia	National registry	Surgical ACL reconstructions	Inpatient + day surgery	High reporting compliance
Canada	Hospital records	ACL procedures (ICD codes)	Inpatient + ambulatory	May vary by province
Nordic countries	National registries	ACL reconstructions	Mostly inpatient	Near-complete coverage; minor variation by country

## Data Availability

The original contributions presented in this study are included in the article. Further inquiries can be directed to the corresponding authors.

## Abbreviations

The following abbreviations are used in this manuscript:

ACL	Anterior cruciate ligament
AAOS	American academy of Orthopedic Surgeons
CNAS	Casa Națională de Asigurări de Sănătate (National Health Insurance House)
ESSKA	European Society of Sports Traumatology, Knee Surgery and Arthroscopy
FIDELITY	Finnish degenerative meniscal lesion study
INSSE	Institutul Național de Statistică (Romanian National Institute of Statistics)
NCSP	Nordic Classification of Surgical Procedures
PCL	Posterior cruciate ligament
COVID-19	Coronavirus disease 2019
MRI	Magnetic Resonance Imaging
